# Allogeneic Hemopoietic Stem Cell Transplantation for Myelofibrosis: 2021

**DOI:** 10.3389/fimmu.2021.637512

**Published:** 2021-05-04

**Authors:** Andrea Bacigalupo, Idanna Innocenti, Elena Rossi, Federica Sora, Eugenio Galli, Francesco Autore, Elisabetta Metafuni, Patrizia Chiusolo, Sabrina Giammarco, Luca Laurenti, Giulia Benintende, Simona Sica, Valerio De Stefano

**Affiliations:** ^1^ Dipartimento di Diagnostica per Immagini, Radioterapia Oncologica ed Ematologia, Fondazione Policlinico Universitario A. Gemelli IRCCS, Roma, Italy; ^2^ Sezione di Ematologia, Dipartimento di Scienze Radiologiche ed Ematologiche, Università Cattolica del Sacro Cuore, Roma, Italy

**Keywords:** myelofibrosis, allogeneic transplantation, busulfan, thiotepa, fludarabine chimerism, splenectomy

## Abstract

The aim of this review is to update the current status of allogeneic hemopoietic stem cell transplants (HSCT) for patients with myelofibrosis (MF). We have first summarized the issue of an indication for allogeneic HSCT, discussing several prognostic scoring systems, developed to predict the outcome of MF, and therefore to identify patients who will benefit of an allogeneic HSCT. Patients with low risk MF are usually not selected for a transplant, whereas patients with intermediate or high risk MF are eligible. A separate issue, is how to predict the outcome of HSCT: we will outline a clinical molecular myelofibrosis transplant scoring system (MTSS), which predicts overall survival, ranging from 90% for low risk patients, to 20% for very high risk patients. We will also discuss transfusion burden and spleen size, as predictors of transplant outcome. The choice of a transplant platform including the conditioning regimen, the stem cell source and GvHD prophylaxis, are crucial for a successful program in MF, and will be outlined. Complications such as poor graft function, graft failure, GvHD and relapse of the disease, will also be reviewed. Finally we discuss monitoring the disease after HSCT with donor chimerism, driver mutations and hematologic data. We have made an effort to make this review as comprehensive and up to date as possible, and we hope it will provide some useful data for the clinicians.

## Indications for HSCT

**Figure 1:**
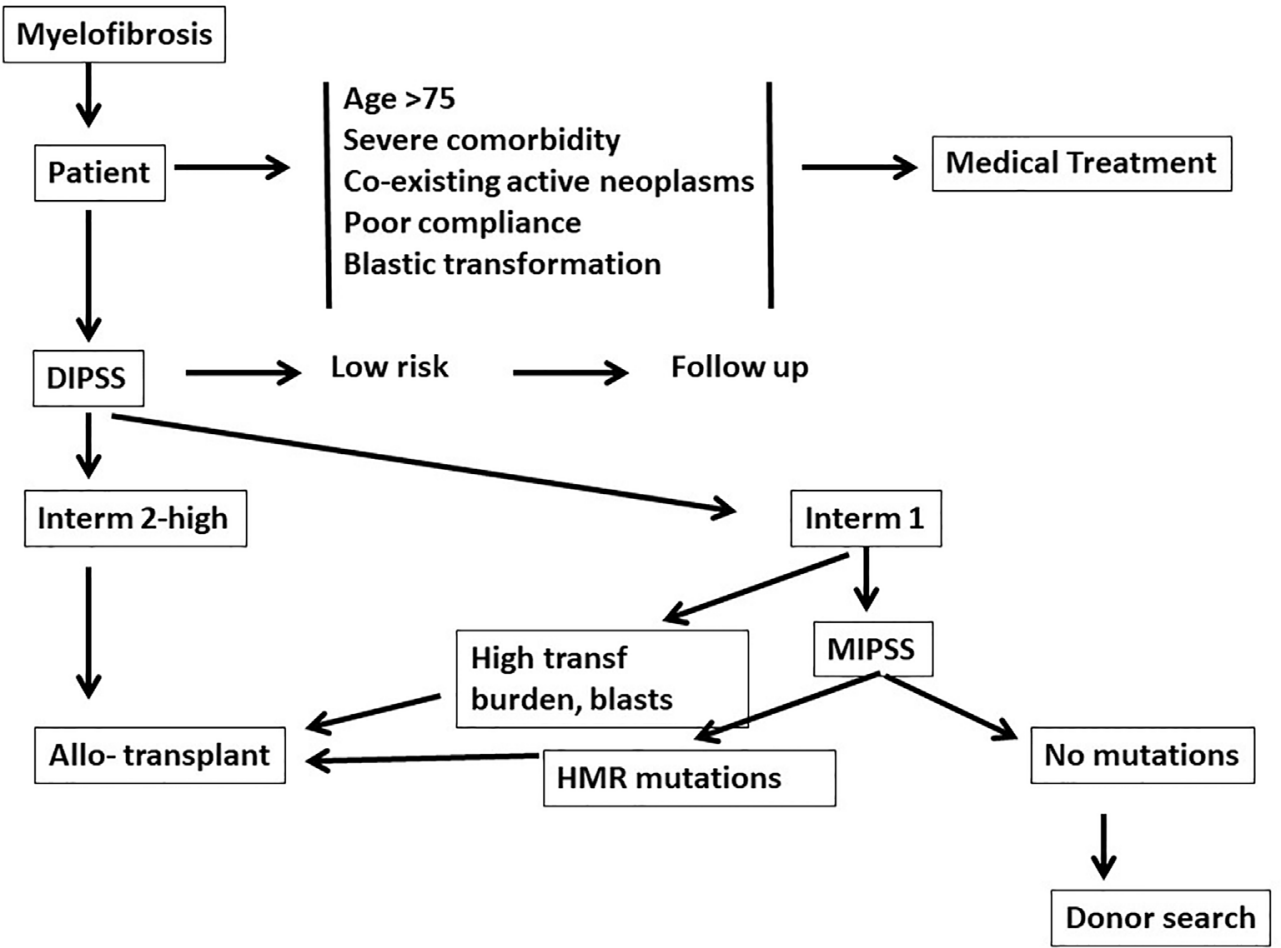
Eligibility for a transplant procedure in patients with myelofibrosis: medical treatment should be offered for older patients (>75 years) and/or patients with comorbidities. Dynamic international prognostic scoring system (DIPSS) will then identify patients low risk patients, who should be followed. DIPSS-intermediate 2 and high risk patients are who are strong candidates for an allogeneic transplant. DIPSS-Intermediate 1 patients with a high transfusion burden and blasts counts are also strong candidates for an allogeneic transplant. Patients may also be studied with a molecular international prognostic scoring system (MIPSS), and may be eligible for transplantation if high risk mutations (HMR) (see text) are identified.

In the era of JAK inhibitors, allogeneic hematopoietic stem cell transplantation (HSCT) remains the only curative treatment for patients with Myelofibrosis (MF) (1). The American Society for Transplantation and Cellular Therapy (ASTCT) considers an allogeneic HSCT “standard of care with clinical evidence” for patients with intermediate and high risk disease (2). In order to classify patients as intermediate or high risk several models have been developed. **Table 1** outlines some of the most commonly used scoring systems and the variables they are based on: IPSS (3), DIPSS (4), DIPSS-plus (5) and MIPSS70 (6). The first two are based exclusively on clinical data, the third incorporates cytogenetics and the fourth includes mutational analysis. Survival of patients with MF can be predicted using one of those models, and thus eligibility for a transplant procedure. However eligibility must also include transplant related variables, such as patients age up to 70-75 years, a good performance status, low transfusion burden, absence of a massive splenomegaly and portal hypertension and donor type. Older patients also tend to have one or more comorbidities which may increase the risk of transplant related mortality (TRM) or even preclude a transplant approach. A Panel of experts recommends considering allogeneic HSCT for patients with IPSS/DIPSS/DIPSS plus high or intermediate-2 risk (7) **(** **Figure 1** **)**. The Panel also recommends considering an allogeneic HSCT for transplant-eligible patients with IPSS/DIPSS/DIPSS-Plus intermediate-1 risk score, who present with either refractory, transfusion-dependent anemia, a percentage of blasts in peripheral blood > 2% in at least two repeated manual measurements, adverse cytogenetics, or high-risk mutations, such as such as ASXL1, EZH2, IDH1/IDH2, SRSF2 (7)**(** **Figure 1** **).** In this situation, the transplant procedure should be performed in a controlled setting (registries, clinical trial) (7).

**Table 1:** Prognostic scoring systems for patients with myelofibrosis.

IPSS [3]	DIPSS [4]	DIPSS-plus [5]	MIPSS70 [6]	
			**Genetic variables:** ✓ One HMR mutation (1 point) ✓ ≥2 HMR mutations (2 points) ✓ Type1/like CALR absent (1 point)	✓
✓ Age > 65 years (1 point) ✓ Constitutional symptoms (1 point) ✓ Hemoglobin < 10 g/dl (1point) ✓ WBC count > 25 × 109/l (1 point) Circulating blasts ≥ 1% (1 point)	✓ Age > 65 years (1 point) ✓ Constitutional symptoms (1 point) ✓ Hemoglobin < 10 g/dl (2points) ✓ WBC count > 25 × 109/l (1 point) Circulating blasts ≥ 1% (1 point)	✓ RBC transfusion (1 point) ✓ PLT count < 100 × 109/l (1 point) Unfavorable karyotype ^a^ (1 point)	**Clinical variables:** ✓ Hemoglobin <10g/dl (1 point) ✓ Leukocytes >25×109/l (2 points) ✓ Platelets <100×109/l (2 points) ✓ Circulating blasts ≥2% (1 point) ✓ Constitutional symptoms (1 point) ✓ Bone marrow fibrosis grade ≥2 (1 point)	
• Low risk: 0 points (11.3 yrs) • Intermediate-1 risk: 1 point (7.9 yrs) • Intermediate-2 risk: 2 points (4 yrs) • High risk: ≥ 3 points (2.3 yrs)	• Low risk: 0 point ( n.r.) • Intermediate-1 risk: 1–2 point (14.2 yrs) • Intermediate-2 risk: 3–4 points (4 yrs) • High risk: 5-6 points (1.5 yrs)	• Low risk: 0 point ( 15.4 yrs) • Intermediate-1 risk: 1 point (6.5 yrs) • Intermediate-2 risk: 2–3 points (2.9 yrs) • High risk: 4–6 points (1.3 yrs)	• Low risk: 0-1 points (n.r.) • Intermediate risk: 2-4 points (6.3 yrs) • High risk: ≥ 5 points (3.1 yrs)	•

IPSS, international prognostic scoring system; DIPSS, dynamic international prognostic scoing system; MIPSS70, mutation-enhanced international prognostic scoring system; HMR, high molecular risk (see text).

More recently a mutation-based prognostic model has been proposed to identify candidates for HSCT among low or intermediate-1 risk DIPSS, who are expected to have similar overall survival as patients with a high risk DIPSS (8). Patients who are triple negative (JAK2/CALR/MPL) or CALR wild type and ASXL1 mutated, irrespective of DIPSS risk scores, should be considered for HSCT (8). A combination of mutation-based prognosis together with clinical data has been compiled in a recent scoring system (9).

In conclusion, we are now able to identify MF patients with a different median survival: there is consensus on the eligibility to transplant for DIPSS intermediate2/high risk patients. The presence of high risk mutations in DIPSS intermediate1/low risk patients may also suggest eligibility for a transplant procedure. The clinical conditions of the patient, the degree of HLA matching of potential donors and the patient’s choice must be considered in the final decision to transplant or not.

## How to Deal With Splenomegaly

Splenomegaly is a common feature in patients with advanced myelofibrosis (MF) and it is a sign of extramedullary hematopoiesis (also known as myeloid metaplasia) (10). Patients may be severely symptomatic with abdominal pain, early satiety, weight loss, cytopenia, portal hypertension, and splenic infarction (10).

**Splenectomy** is effective in relieving symptoms, but is associated with a number of complications, as well as significant morbidity and mortality.

Peri-operative mortality is in the range of 5%-10%. The most common complications are infections, thrombosis and bleeding, occurring in up to 30% of patients (11). Patients with thrombocytopenia seemed to have an increased probability of post-splenectomy blast transformation, although this did not result in shortened survival. Leukemic transformation is more probably related to natural progression of the disease in advanced stage and to post-splenectomy redistribution of circulating blasts, not to true clonal evolution (12, 13). Hemopoietic stem cell transplantation (HSCT) offers the potential of cure for patients with intermediate or high risk myelofibrosis (14). Splenomegaly, characteristic of those patients, may lead to sequestration of transplanted stem cells and delayed hematologic recovery (3, 15) thus affecting the transplant outcome. Surgical removal of the spleen may be effective in reducing the time for neutrophil and platelet recovery (16) but its impact on relapse rate and survival is unclear (17, 18), calling for a prospective randomized trial. Pre-transplant splenectomy in MF patients was associated with a prolonged overall and event-free survival in a recently published study (19).

The advent of **Janus kinase (JAK) 1/2 inhibitors**, which decrease splenomegaly and alleviate MF-related symptoms, has had, as compared to old cytoreductive drugs, a major impact on the management of splenomegaly, removing some indications for splenectomy. However, in a proportion of patients, the splenic response is then lost. Many MF patients who proceed to allogeneic HSCT, are currently treated with JAK inhibitors, usually ruxolitinib: this should be tapered down over a 10- to 14-day period and should be discontinued just before the conditioning regimen (20). In one study, ruxolitinib was continued also during transplant in the attempt of preventing GvHD (21).

**Splenic irradiation (SI)** may also be used to reduce the spleen size and related symptoms; there are only few small studies on SI prior to transplant in MF patients (22, 23). It was demonstrated that SI alleviates splenic discomfort and reduces spleen size in a majority of MF patients, with a median duration of response of 6 months (24). Limitations of SI include prolonged pancytopenia with infectious complications. Comparable engraftment rate has been shown in patients receiving or not SI (25) as well as comparable acute and chronic GVHD incidence, post-transplant infectious complications and survival. The role of SI in leukemic transformation (LT) remains unclear and speculative. Radiotherapy may be indicated in patients who are not eligible for surgery or in patients who have lost their response to JAK2 inhibitors (26–28).

## Predicting the Outcome of HSCT

**Disease based risk score.** Survival of MF patients receiving medical treatment, with the exclusion of allogeneic HSCT, can be predicted by several scoring systems, reviewed in Indications for HSCT (3–7). Some studies have assessed whether these scoring systems can predict the outcome of patients after an allogeneic HSCT. DIPSS can predict post transplant survival (29), and the same has been shown for DIPSS-plus (30). In multivariate analysis, the DIPPS-plus score predicted survival, disease free survival (DFS) and TRM, together with conditioning regimen, comorbidity index (HCT-CI), patients’ age and donor type (30). In 2019, a cohort of 159 patients with secondary myelofibrosis who underwent allogeneic HSCT was analyzed retrospectively to compare the predictive value of DIPSS and MYSEC (31). The four risk groups of DIPSS did not predict survival after allogeneic HSCT, whereas MYSEC maintained its predictive role also in the post-transplant setting.

**Transplant based risk score (TS).** Few scoring systems have been designed exclusively for allogeneic HSCT. In 2010, a study identified spleen size, transfusion history and donor type as predictive of outcome: survival was 79% for low risk patients and 8% for high risk patients (18). In 2012 a predictive risk model including JAKV617F status, age and constitutional symptoms was proposed in the setting of 150 transplanted patients and resulted to be predictive for 5 years overall survival (OS) (32).

More recently, a scoring system has been devised, which incorporates HLA matching between donor and recipient, mutational analysis, and clinical data, at time of transplantation (MTSS), in patient with primary and secondary MF (9). This index is predictive of non-relapse mortality. In the last year we have revisited our transplant score (TS) including maximum spleen size and red blood cell transfusion burden before HSCT (18): the 5 year disease free survival (DFS) was 74% *vs* 36% (p=0.0001) for patients with low or high TS.

In conclusion, scoring systems designed to predict transplant outcome are available and can be used when counseling patients eligible for transplant procedures.

## Donor Type, Stem Cell Source and GvHD Prophylaxis

Donor type is an important predictor of outcome in myelofibrosis: a study from the Center for International Blood and Marrow Transplant Research (CIBMTR) on 233 transplants for myelofibrosis (33), showed that donor type was an independent risk factor for TRM, with a relative risk of death of 3.92 for matched unrelated donor (MUD) and 9.37 for mismatched unrelated donor (MMUD), when compared to matched related donor (MRD) (33). The 5 year overall survival was 56% for MRD, 48% for MUD and 34% for MMUD. The main causes of death were GvHD, infections and organ failure, in particular among MMUD grafts (33). Similar results are reported in other studies (17, 34–37). On the other hand, contrasting data exist regarding GvHD and donor type. Some studies show no significant difference among different donor types (17, 35, 38), whereas the CIBMTR shows a higher risk of GvHD for patients receiving MUD (RR 1.98) and MMUD (RR 1.52) as compared to MRD (33). Engraftment is reported to be comparable according to donor type (33, 35, 38), whereas significant differences have been described according to the stem cell source, with faster recovery with peripheral blood grafts (37, 38). Unrelated cord blood (UCB) transplants have been rarely used in myelofibrosis, and are associated with delayed engraftment and a high TRM, probably due to the significant risk for graft failure and infectious complications (39).

The addition of ATG to conventional GvHD prophylaxis, based on calcineurin inhibitor alone or combined to methotrexate or mycophenolic acid, reduces the incidence of GvHD, as one would expect (40). However, modified regimens of GvHD prophylaxis, including the use of post-transplant cyclophosphamide (PT-CY) have reduced post-transplant complications in alternative donor grafts, especially for HLA haplo-identical donor (36). A combination of calcineurin inhibitor with ATG and PTCy after reduced intensity conditioning may further reduce the risk of GvHD, improving TRM and survival, without an increased risk of relapse (41). Very recently, an interesting pilot study was conducted by Morozova and colleagues: GvHD prophylaxis with PTCY and ruxolitinib showed promising results in terms of GvHD control in a small cohort of patients with acceptable TRM (42).

In summary an HLA matched donor is the best option for myelofibrosis, in order to achieve optimal outcome: alternative donor grafts may be explored using modified regimens of GvHD prophylaxis.

## Conditioning Regimens and Ruxolitinib

Conditioning regimens in myelofibrosis were historically myeloablative (MAC), predominantly busulfan plus cyclophosphamide and total body irradiation with or without cyclophosphamide (15), but transplant related mortality (TRM) and GvHD rates were high, especially in older individuals (43).

Reduced intensity conditioning (RIC) has been increasingly used in MF, in consideration of the older age of MF patients. The first prospective EBMT multicenter phase II trial of RIC SCT consisted of busulfan (10 mg/kg) orally (or equivalent IV dose) plus fludarabine (180 mg/m^2^) and *in vivo* T-cell depletion with anti-thymocyte globulin at a dose of 3 x 10 mg/kg (for related transplantation) or 3 x 20 mg/kg (for unrelated donor transplantation): this protocol resulted in low rates of primary graft failure and rapid hematologic recovery (17). Fludarabine 90 mg/m^2, combined with melphalan 140 mg/m^2 (FLU-MEL) is an alternative RIC regimen, and has been compared in a retrospective study with the BU-FLU regimen (44). Although the FLU-MEL was associated with increased early toxicity, the long-term outcome (OS and disease-free survival) was similar in the two groups. In both regimens the use of a HLA mismatched unrelated donor was associated with worse outcome, in terms of TRM, OS and progression-free survival. A randomized study comparing fludarabine in combination with busulfan 10 mg/kg i.v. or thiotepa 12 mg/kg, failed to identify significant differences in terms of clinical outcome (45): both regimens were associated with a significant degree of mixed chimerism.

In a retrospective comparisons of RIC versus MAC regimens for myelofibrosis, the latter do not appear to protect patients from relapse (46), neither there are differences within day +100 transplant-related mortality (47). A large retrospective analysis of the EBMT in 2224 patients with myelofibrosis, compared MAC regimens (781 patients) with RIC regimens (1443 patients) (48): there was no statistically significant difference in engraftment, GvHD, TRM and overall survival; there was a trend toward a higher relapse rate with RIC.

We have recently shown that a conditioning regimen including two alkylating agents (in our case busulfan and thiotepa) with fludarabine, significantly reduced the risk of relapse when compared to regimen with one alkylating agent (either busulfan or thiotepa or melphalan) in combination with fludarabine (36). Therefore, the choice of the conditioning regimen, may play a significant role in determining the control of the disease after an allogeneic HSCT.

The efficacy of the JAK1/JAK2 inhibitor ruxolitinib in reducing spleen size and systemic symptoms, in myelofibrosis, has been established (49, 50). Currently, most patients undergoing an allogeneic HSCT have been treated with this agent with the aim of reducing splenomegaly, improving the performance status and shorten time to engraftment. A phase II trial demonstrated the feasibility of ruxolitinib therapy followed by a RIC regimen for patients with myelofibrosis (51). Appropriate tapering should be scheduled (52), although recently peri-transplant ruxolitinib has been reported (42). There is no evidence, however, that the administration of ruxolitinib pre-transplant reduces the incidence of relapse after transplant.

## Monitoring Disease Control (Donor Chimerism and Mutations)

Patients with myelofibrosis may have one of three driver mutations (JAK2, CALR and MPL), or lack all three (triple negative patients). Ditschkowski et al. (53) showed that survival after transplantation was not significantly different for JAK2+ (75%) versus JAK2 negative (71%) patients. More recent retrospective studies have suggested a survival advantage for CALR mutation (54, 55). A large retrospective study has investigated the role of extensive mutational profiling with a targeted 16-gene panel, and has confirmed the favorable role of a CALR mutation (56). In the same study IDH2 and ASXL1 mutations confirmed their adverse prognostic role after allogenic HSCT, whereas a triple negative status (JAK2, MPL, CALR) did not appear to modify the outcome after transplant.

Minimal residual disease (MRD) should be used to identify patients achieving a complete remission after HSCT, as well as an early evidence of relapse. Alchalby et al. has shown that JAK2 negativity after allogeneic HSCT significantly reduces the risk of relapse (57). Similar results have been obtained with MPL and CALR mutations as MRD markers (58). A recent retrospective single-center study (59) has shown that that patients with detectable mutations on day +100 or at day +180 after allogeneic HSCT have a significant higher risk of clinical relapse at 5 years, as compared to molecular-negative patients (62% *vs* 10%, P<0.001 and 70% *vs* 10%, P<0.001, respectively): single different mutations have comparable predictive value on relapse.

However, 10% to 15% of patients are triple negative and cannot be followed after transplantation with a molecular marker: in these patients chimerism studies can be helpful to identify early signs of relapse. We have recently described 120 patients with chimerism data on day +30 (60), showing that early full donor chimerism is highly predictive of long-term disease control. The cumulative incidence of relapse at 5 years, was 14% *vs* 40% for patients with or without full donor chimerism (40). We found that a conditioning regimen including two alkylating agents (busulfan and thiotepa) induces a significantly higher rate of complete donor chimerism on day +30, as compared to patients prepared with one alkylating agent (either busulfan, melphalan or thiotepa) (87% *vs* 45%, p<0.0001).

MRD positive patients or patients with declining donor chimerism, who still are receiving immunosuppressive therapy, may discontinue immunosuppressive drugs and/or receive donor lymphocyte infusions (DLI), in order to achieve again full donor chimerism.

## Primary Graft Failure (PrGF) and Poor Graft Function (PGF)

Lack of engraftment of donor stem cells is referred to as primary graft failure (PrGF), and is characterized by neutropenia, combined with mixed or no donor chimerism on bone marrow and/or peripheral blood cells (61). PrGF should be distinguished from poor graft function, or cytopenia with full donor chimerism (62). The latter suggests inappropriate function of engrafted donor stem cells and can be treated with the infusion of selected CD34+ cells from the same donor, without a preparative regimen (62). Predictive factors have not been determined, but several conditions have been associated with unsuccessful engraftment, such as the intensity of the conditioning regimen, donor type, stem cells source, number of CD34+ cells infused, GvHD prophylaxis, degree of fibrosis, degree of splenomegaly, pre-transplant thrombocytopenia (63).

The incidence of PrGF ranges from 2 to 24%. A lower rate was reported in a large prospective study from EBMT (48), with only in 2 out 103 patients with PrGF. However, 11% of patients experienced poor graft function and required an additional stem cell boost. In a subsequent pilot study, PrGF was not influenced by the intensity of conditioning regimen (64) and no other predictors were found in other studies (17, 53). Donor type appears to influence the incidence of PrGF, which is lower in patients transplanted from HLA identical donors, as compared to transplants from family mismatched and unrelated donors (65–67). Contrasting data are reported on other factors: splenectomy before HSCT, peripheral stem cell use as source of stem cells and the absence of pre-transplant thrombocytopenia have been suggested to promote engraftment in some studied (18, 66, 68), but not in other studies (65).

Patients with full donor engraftment, may still have transfusion dependent low blood counts for variable periods of time, and this is referred to as Poor graft function (PGF). In a large retrospective analysis, the proportion of patients with less than 20x10^9/l platelets between day +50 and +100 after an allogeneic HSCT, is 10% and has not changed in the time period before 2000, 2001-2010 and beyond 2010 (unpublished). A diagnosis of myelofibrosis is a negative predictor for hematologic recovery: a low platelet count is seen in 18% *vs* 8% of patients with or without a diagnosis of MF (unpublished). For this reason, when looking at patients receiving a top up of CD34 selected cells for PGF, the proportion of patients with MF (26%) is higher than the proportion of MF in the transplant indications (7%) (62). These patients may remain transfusion dependent for long periods of time, and may be treated either with an infusion of CD34 selected cells from the same donor, or, more recently with high dose eltrombopag. Time to trilineage recovery is however delayed with these approaches and long-lasting supportive care must be planned.

## Treatment of MF Relapse After Allogeneic Transplant

Allogeneic hematopoietic stem cell transplant remains the only curative treatment for myelofibrosis (MF). A retrospective EBMT study on 1055 patients with MF transplanted between 1995 and 2014, alive and free of their disease at two years after HSCT showed that the most common cause of death (41-61%) was relapse of MF, for all time periods (2-5years, 5-10 years) (40). There is no standardized re-treatment of relapse after allogeneic transplant. Based on limited available literature, ruxolitinib, donor leukocytes infusion (DLI), and a second allogenic HSCT are three options for relapsing MF patients; obviously, the choice depends on patients age, fitness status, molecular or hematologic relapse, and the presence of GVHD.

The use of DLI and second transplant as salvage treatment for relapsed MF after allogeneic HSCT was reported in a retrospective study some years ago (69). Out of 26 relapsed patients, 39% achieved a stable response to dose-escalated DLIs. Seventeen patients, thirteen of which non-responders to DLI, underwent a second allogeneic HSCT, achieving an ORR of 80% (9 CR and 3 PR); incidence of relapse at 1-year was 24%. The 2-year overall survival and progression-free survival were 70% and 67%, respectively.

The most consistent data derive from a recent EBMT real-life retrospective study focusing on the treatment of 251/1371 (18%) MF patients, who relapsed after an allogeneic HSCT (70). DLIs were used in 23% of patients, whereas 20% underwent DLI combined with chemotherapy and 11% had chemotherapy alone. Fifty-one patients (25%) underwent second allogeneic HSCT alone and 26 (13%) underwent DLI and a second allogeneic HSCT. The median OS from the time of relapse for patients receiving DLI alone, DLI followed by a second allogeneic HSCT or second allogeneic HSCT were 76 months, 54 months, and 27 months respectively.

Recently Chabra et al. published a small number of MF patients, mostly treated with ruxolitinb pre-transplant (71): after a median follow up of >3 years, two patients out of 37 had relapsed after HSCT (5.4%), but the study lacked a strong control group of untreated ruxolitinib patients. Indeed other recently published data in the ruxolitinib era (72), have shown no improvement in survival nor in the incidence of relapse for MF. The use of ruxolitinib after allogenic HSCT is primarily attributable to the treatment of GVHD, and only in few cases for the treatment of the relapse, mostly in combination with DLIs. One study has reported peri-transplant use of ruxolitinib (21).

In conclusion, although based on a small number of studies, the best therapeutic strategy for MF patients relapsing after an allogeneic HSCT, seems to be dose -escalated DLI, or otherwise, for non-responders, a second allogeneic HSCT. The question remains whether DLI should be infused after a lympho-depleting treatment, as currently is being done for CAR-T cells.

## Designing a Transplant Strategy for Myelofibrosis

Patients with myelofibrosis need to be discussed to identify eligibility for transplant procedures **(** **Figure 1** **).** Patients over the age of 75 years, with severe comorbidities, coexisting active neoplasms, or poor compliance, should be addressed by medical treatment. Patients less than 75 years of age and fit, should be assessed for risk factors (DIPSS or other scoring systems): low risk patients should be followed regularly. DIPSS intermediate 2 or high risk patients are eligible for a transplant procedure **(** **Figure 1** **).** DIPSS int 1 patients should be studied with next generation sequencing (NGS): if no additional adverse mutations are found (ASXL1, EZH2, SRSF2, IDH1/2) then the search for a donor can be initiated, but the transplant may be postponed. If, on the contrary, additional adverse mutations are identified the donor search may be initiated and the transplant also programmed.

Once a transplant is programmed several facts need to be considered: in addition to patient factors such as age, comorbidities and disease phase (DIPSS), other facts need to be taken in to account, including transplant variables (donor type, stem cell source, conditioning regimen, GvHD prophylaxis), the psychological status of the patient, the presence of care givers, especially for the post-transplant discharge and logistics (transplant centers may be located at a distance from the patients’ home). The combination of all these factors will then lead to a tailored strategy in terms of optimal timing and choice of a transplant platform.

## Author Contributions

AB, SS and VS designed the study and overviewed the manuscript. Sections and authors: indications (II), splenectomy (ER), predicting outcome (FS, EG), monitoring disease (PC), Graft Failure (SG), Relapse (LL), Conditioning regimens (FA), donor type (EM), reviewed MS (GB). All authors contributed to the article and approved the submitted version.

## Funding

This study was partly funded by AIRC, Associazione Italiana Ricerca contro il Cancro; grant to AB.

## Conflict of Interest

The authors declare that the research was conducted in the absence of any commercial or financial relationships that could be construed as a potential conflict of interest.
